# Identification of potential molecular pathways involved in prostate carcinogenesis in offspring exposed to maternal malnutrition

**DOI:** 10.18632/aging.104093

**Published:** 2020-10-13

**Authors:** Sérgio Alexandre Alcantara Santos, Ana Carolina Lima Camargo, Flávia Bessi Constantino, Ketlin Thassiani Colombelli, Luiz Marcos Frediani Portela, Matheus Naia Fioretto, José Cavalcante Souza Vieira, Pedro Magalhães Padilha, Mateus Betta de Oliveira, Sergio Luis Felisbino, Robson Francisco Carvalho, Luis Antonio Justulin

**Affiliations:** 1 Department of Structural and Functional Biology, Institute of Biosciences, Sao Paulo State University (UNESP), Botucatu 18618-689, São Paulo, Brazil; 2 Department of Chemical and Biological Sciences, Institute of Biosciences, Sao Paulo State University (UNESP), Botucatu 18618-689, São Paulo, Brazil

**Keywords:** DOHaD, prostate diseases, maternal exposure to low protein diet, mass spectrometry

## Abstract

The developmental origins of health and disease concept links adult diseases with early-life exposure to inappropriate environmental conditions. Intrauterine and postnatal malnutrition may lead to an increased incidence of type 2 diabetes, obesity, and cardiovascular diseases. Maternal malnutrition (MM) has also been associated with prostate carcinogenesis. However, the molecular mechanisms associated with this condition remain poorly understood. Using a proteomic analysis, we demonstrated that MM changed the levels of proteins associated with growth factors, estrogen signaling, detoxification, and energy metabolism in the prostate of both young and old rats. These animals also showed increased levels of molecular markers of endoplasmic reticulum function and histones. We further performed an *in silico* analysis that identified commonly deregulated proteins in the ventral prostate of old rats submitted to MM with a mouse model and patients with prostate cancer. In conclusion, our results demonstrated that estrogenic signaling pathways, endoplasmic reticulum functions, energy metabolism, and molecular sensors of protein folding and Ca2+ homeostasis, besides histone, and RAS-GTPase family appear to be involved in this process. Knowledge of these factors may raise discussions regarding the role of maternal dietary intervention as a public policy for the lifelong prevention of chronic diseases.

## INTRODUCTION

Advances in public health and preventive medicine have resulted in an unprecedented and welcomed number of individuals reaching old age. Despite longevity, there has been observed an increase in older populations affected by chronic diseases that demand specialized and expensive elderly care services. The identification and widespread public awareness of unhealthy modifiable risk factors such as western diet consumption and obesity, sedentary lifestyle, smoking, stress, and insufficient sleep, followed by the adoption of a healthy lifestyle is a feasible, safe, and effective low-cost public policy program to improve the quality of life with aging [1, 2]. Intrauterine and early postnatal life experiences may permanently modulate health trajectories across the lifespan [3–5]. The hypothesis that the intrauterine period of development may modulate offspring postnatal health was initially proposed by David Barker in "Fetal Origin of Adult Diseases" (FOAD), almost 30 years ago [6]. Subsequently, FOAD evolved to consolidate the "Developmental Origins of Health and Disease" (DOHaD) concept by including both pre-conception and early postnatal life as a new window of susceptibility. Recently, epigenetics has become one of the most relevant molecular mechanisms associated with the transgenerational inheritance involved with DOHaD [7].

Despite the difficulty in confirming the impact of maternal and early life adversity on human health, some tragic events in human history were crucial in supporting the DOHaD concept. For example, the "Dutch Hunger Winter" was a period of severe famine in the western part of the Netherlands at the end of World War II and has provided an opportunity to explore the effects of intrauterine malnutrition on subsequent adult health [8]. First published in 1976, the Dutch Hunger Winter Cohort has been explored to confirm the developmental origin of non-communicable chronic diseases, e.g., cardiovascular disease, obesity, type 2 diabetes, schizophrenia, and infertility in the progeny exposed to famine [9–11]. Importantly, epidemiological and experimental studies have also supported the DOHaD concept as a mechanistic framework related to early life carcinogenesis (such as breast and prostate cancer) [12–17]. Among the malignancies affecting men, prostate cancer (PCa) is the second most diagnosed cancer worldwide. In 2018, the Global Cancer Statistics (GLOBOCAN) estimated almost 1.3 million new cases of PCa globally, leading to 359,000 deaths [18]. Although multifactorial etiology, genetic background, ethnicity, and aging are consistently established risk factors for PCa. However, evidence supporting the early origin of PCa is growing. William Gardner (1995) proposed, almost 30 years ago, the "Prenatal origin of PCa" hypothesis [19]. After that, some epidemiological studies have reinforced Gardner's hypothesis on the early life origins of PCa, as diagnosed in older men [14, 20, 21]. These authors proposed that exposure to certain environmental conditions during pregnancy, such as malnutrition or chemical endocrine disruptors, may alter maternal steroid hormone profiles, thereby modifying the offspring's PCa risk throughout life. This effect was consistently observed in African American men, who are at high risk for PCa, and whose mothers have higher levels of estrogen during pregnancy compared to Caucasian women [20].

In one of the few opportunities to explore how exposure to adverse conditions during windows of vulnerability interferes with human PCa, Keinan-Boker et al. [13] demonstrated that Jewish men exposed during early life to famine and stress during the Holocaust were at a higher risk for several types of cancer (including PCa) later in life. Similarly, women severely exposed to famine during the Dutch hunger winter were at increased risk for breast cancer development [22]. Interestingly, a higher risk for breast cancer was observed for women who were exposed to famine between the ages of 2 and 9 years. Dirx et al. [23] analyzing data from the Netherlands Cohort Study, showed a slight increase in PCa risk among men exposed to famine during adolescence (a critical window of vulnerability for reproductive organs) compared with those men living in northern and southern parts of the Netherlands, who had almost no exposure to famine. These data reinforce the need to explore, in-depth, the potential of early life malnutrition as an environmental risk factor for prostate carcinogenesis across the lifespan.

Emerging experimental studies have been designed to explore the potential of early life exposure to environmental risk factors on prostate carcinogenesis. The intrauterine or neonatal exposure to endocrine disruptors, such as phthalate or bisphenol A, has been associated with the deregulation of critical molecular pathways involved in prostate carcinogenesis in rat offspring [17, 24, 25]. Regarding maternal malnutrition (MM), Santos et al. [16] demonstrated that offspring born from dams fed with a low protein diet (LPD) during gestation and lactation were at high risk of developing prostatic disorders with aging, including carcinoma *in situ* in the ventral prostate (VP) lobe. Overall, it has been proposed that early life exposure to endocrine disruptors or malnutrition may show early deregulation and persistent cellular response to estrogen signaling pathways, probably involving changes in epigenetic markers, such as DNA methylation and the expression of microRNAs, leading to an increased incidence of prostatic disorders with aging [17, 25–27].

Although MM can be identified as a potentially modifiable environmental risk factor for offspring prostate carcinogenesis, there is a lack of information regarding the molecular mechanisms involved in this process. We previously demonstrated, for the first time, that maternal exposure to a low protein diet promotes prostate carcinogenesis in older rat offspring [16]. In the current investigation, we used mass spectrometry to identify, in young and older offspring, changes in the proteomic profile potentially involved in the early life origins of prostate carcinogenesis observed with aging.

## RESULTS

### Maternal LPD reduced weight gain and imbalance of steroid hormones in male offspring

**Figure 1:**
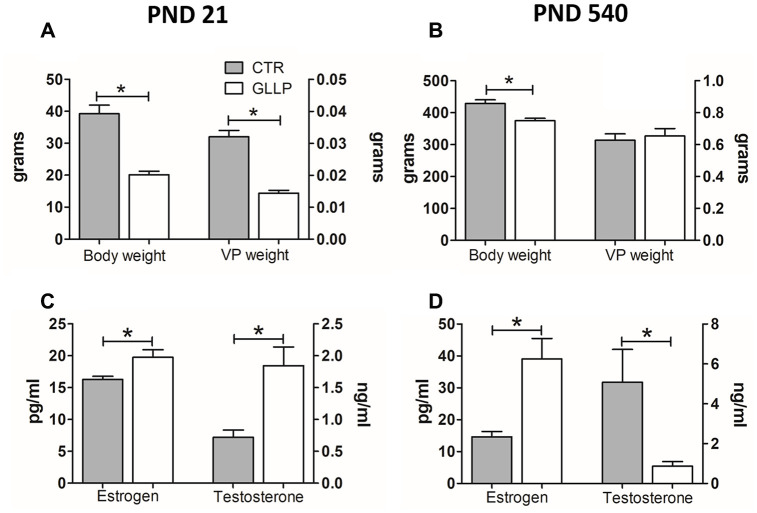
Body weight (**A**, **B**) and hormonal levels (**C**, **D**) of male offspring on PND 21 and 540. All data are expressed as mean±SD. Asterisks (*) represent statistical differences between experimental groups with p<0.05. CTR = control; GLLP = gestational and lactational low protein; PND = postnatal day; VP = ventral prostate.

Offspring body weight was lower in the LPD animals on both postnatal days (PND) 21 and 540 compared to the respective control (CTR) groups (Figure 1A, 1B). The serum steroid hormones estrogen (17β-estradiol) and testosterone (17β-hydroxy-4-androstene-3-one) increased in the GLLP group on PND 21 compared to the CTR group (Figure 1C). However, on PND 540, while estrogen was higher in the GLLP group, testosterone levels decreased compared to the CTR group, leading to an increased estrogen/testosterone ratio (Figure 1D).

### Early and late effects of maternal LPD on offspring VP

**Figure 2:**
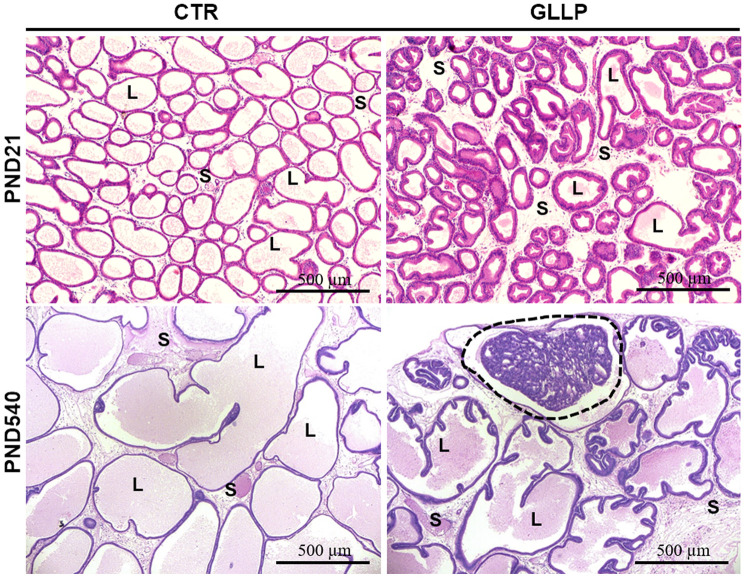
**Representative histological sections of the VP lobes from the CTR and GLLP groups on PND 21 and 540, stained with hematoxylin-eosin (HE).** Glandular growth in the GLLP group on PND 21 was impaired compared to the CTR. At PND 540, the carcinoma in situ was highlighted by the dashed circle. S = Stroma, L = Lumen, E = Epithelium, Scale bar: 500 μm.

On PND 21, the morphological analyses demonstrated an impairment of prostate growth in the GLLP group, characterized by a smaller prostatic secretory structure, reduced luminal compartment, and increased epithelial and stromal compartments, compared to the CTR group (Figure 2A, 2B). On PND 540, while we did not identify carcinoma in the CTR group (Figure 2C), the histopathological analysis confirmed the presence of carcinoma *in situ* in the animals from the GLLP group selected for a mass spectrometry analysis (Figure 2D).

### Maternal LPD changed the proteomic profile in the prostate offspring at both ages

**Figure 3:**
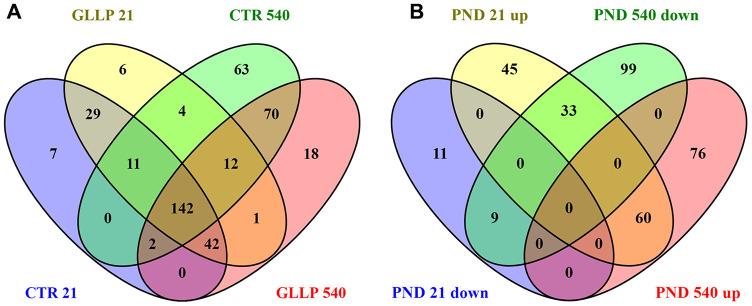
**Venn diagram.** (**A**) Shared proteins between CTR and GLLP groups in PND 21 and 540. (**B**) Shared proteins differentially expressed between the CTR and GLLP groups on PND 21 and 540. CTR = Control; GLLP = Gestational and lactational low protein; PND = Postnatal day; up = upregulated proteins; down = downregulated proteins.

Figure 3 shows a total of 256 proteins identified in the VP by MS/MS approach on PND 21. Of these, 158 proteins were significantly differentially expressed in the GLLP group compared to the CTR group, including 138 and 20 proteins that were up- and downregulated, respectively. On PND 540, 366 proteins were significantly differentially expressed in the GLLP group compared to the CTR group, including 135 and 141 proteins that were up- and downregulated, respectively. The complete list of proteins is described in Supplementary File 1.

### Functional enrichment identified molecular pathways altered by maternal LPD in the offspring prostate

**Figure 4:**
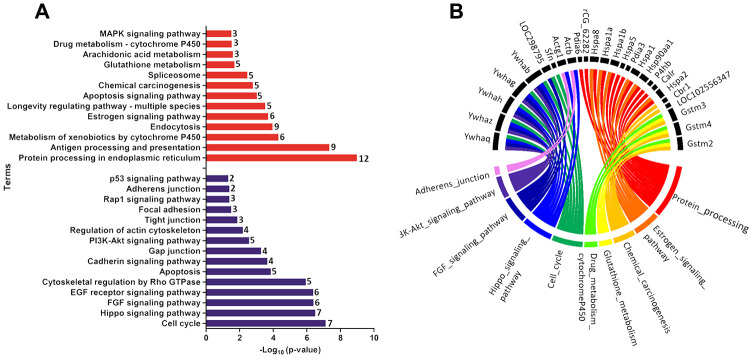
(**A**) Ontological enrichment of upregulated (red) and downregulated (blue) proteins on PND 21 by KOBAs 3.0. All data were expressed as -Log10 (p-value). (**B**) Circus plot graphic identifying the top 10 enriched terms and the DEP associated with each term. The numbers in front of the bars mean the number of proteins that enriched each term.

**Figure 5:**
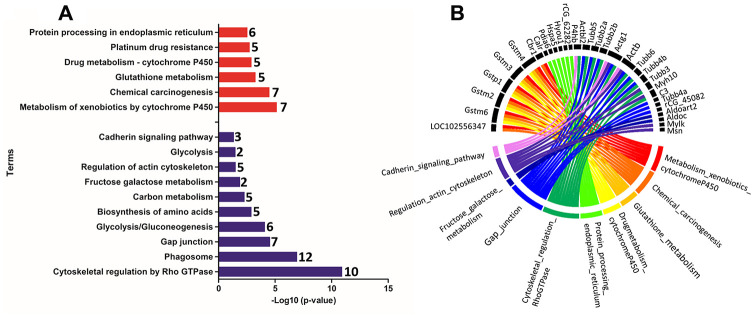
(**A**) Ontological enrichment of upregulated (red) and downregulated (blue) proteins on PND 540 using the KOBAs 3.0 tool. All data were expressed as -Log10 (p-value). (**B**) The Circus plot graphic identifying the top 10 enriched terms and the DEP associated with each term.

Functional enrichment was performed for the set of downregulated and upregulated proteins separately in both ages. The red and blue bars in Figure 4A demonstrate enriched terms for up and downregulated proteins on PND 21, respectively. Upregulated proteins enriched terms related to protein processing in the endoplasmic reticulum, antigen processing and presentation, metabolism of xenobiotics by cytochrome P450, endocytosis, estrogen signaling pathway, longevity regulating pathway - multiple species, apoptosis signaling pathway, chemical carcinogenesis, spliceosome, glutathione metabolism, arachidonic acid metabolism, drug metabolism - cytochrome P450, and MAPK (mitogen-activated protein kinase) signaling pathways. Downregulated proteins enriched terms related to cell cycle, Hippo signaling pathway, FGF (fibroblast growth factor) signaling pathway, EGF (epidermal growth factor) receptor signaling pathway, cytoskeletal regulation by Rho GTPase, apoptosis, cadherin signaling pathway, gap junction, phosphatidylinositol 3' -kinase (PI3K)-AKT signaling pathway, regulation of actin cytoskeleton, tight junction, focal adhesion, RAP1 (Ras-proximate-1) signaling pathway, adherents junction, and p53 signaling pathway. Using a circus plot analysis, we identified the set of deregulated proteins (up and down) associated with each enriched term on PND 21 (Figure 4B). On PND 540, the upregulated proteins enriched terms related to the metabolism of xenobiotics by cytochrome P450, chemical carcinogenesis, glutathione metabolism, drug metabolism - cytochrome P450, platinum drug resistance, and protein processing in the endoplasmic reticulum. Downregulated protein-enriched terms related to cytoskeletal regulation by Rho-GTPase, phagosome, gap junction, glycolysis/gluconeogenesis, biosynthesis of amino acids, carbon metabolism, fructose galactose metabolism, regulation of actin cytoskeleton, glycolysis, and cadherin signaling pathway (Figure 5A). Using a circus plot analysis, we identified the set of deregulated proteins (up and down) associated with each enriched term on PND 540 (Figure 5B). The list of proteins that enriched each molecular term is described in Supplementary File 2.

**Figure 6:**
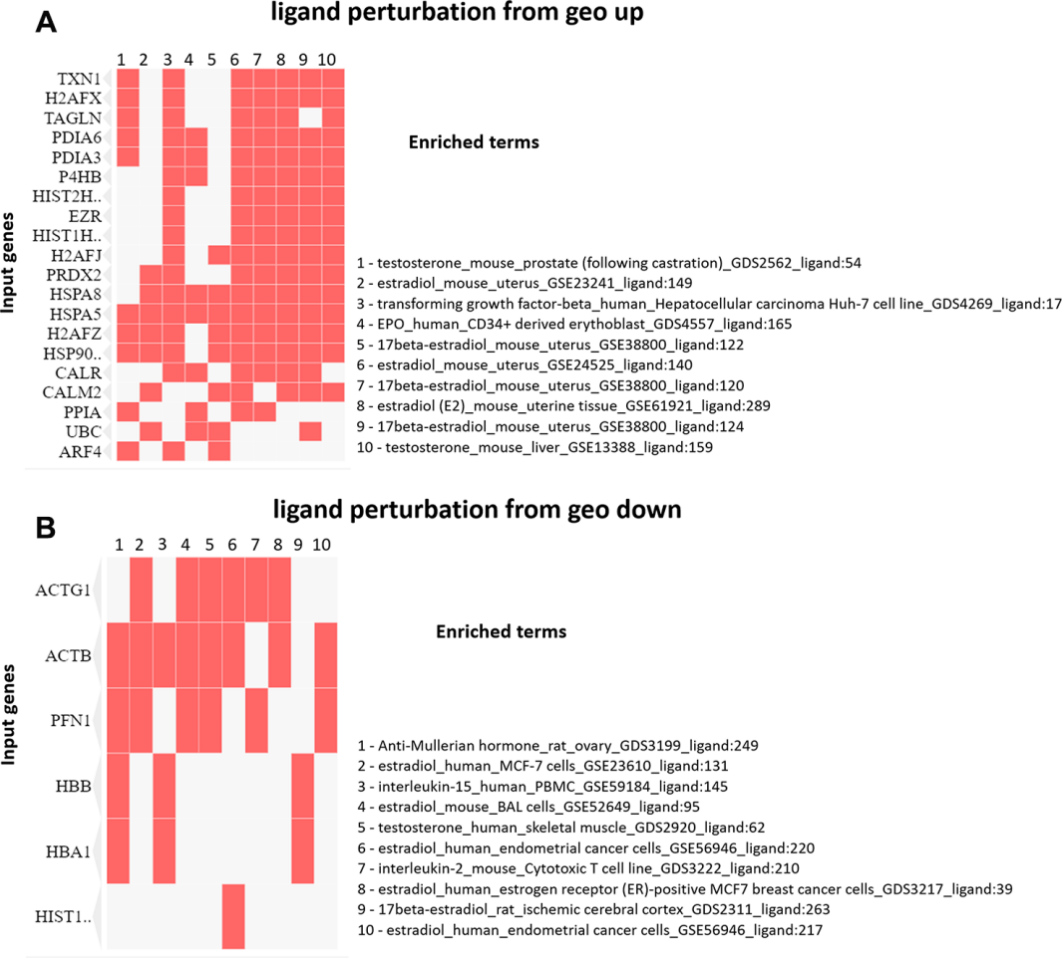
(**A**) Clustergram generated by Enrichr using upregulated proteins on PND 21. The red cells in the matrix indicate the genes associated with each term. It was demonstrated the top 10 enriched terms with p-value <0.05. (**B**) Clustergram generated by Enrichr using downregulated proteins on PND 21. The red cells in the matrix indicate the genes associated with each term. It was demonstrated the enriched terms with p-value <0.05 (top 10).

**Figure 7:**
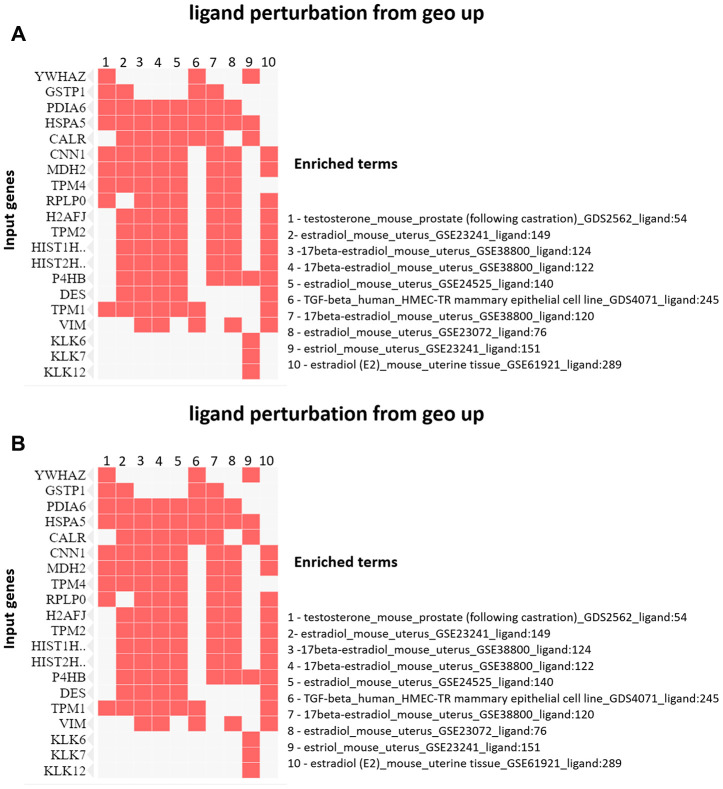
(**A**) Clustergram generated by Enrichr using upregulated proteins on PND 540. The red cells in the matrix indicate the genes associated with each term. It was demonstrated the enriched terms with p-value <0.05 (top 10). (**B**) Clustergram generated by Enrichr using downregulated proteins on PND 540. The red cells in the matrix indicate the genes associated with each term. It was demonstrated the enriched terms with p-value <0.05 (top 10).

The enrichment analysis from Ligand Perturbation UP/DOWN database showed that upregulated proteins (in both PND 21 and 540) are associated with hormonal treatment, especially testosterone and estrogen (Figure 6A, 6B). The set of downregulated proteins for both ages also enriched terms related to exposure to testosterone and estrogen (Figure 7A, 7B). Overall, these results highlighted the involvement of a hormonal imbalance on maternal LPD-induced prostate disorders in offspring.

### Protein-Protein Interaction network

**Figure 8:**
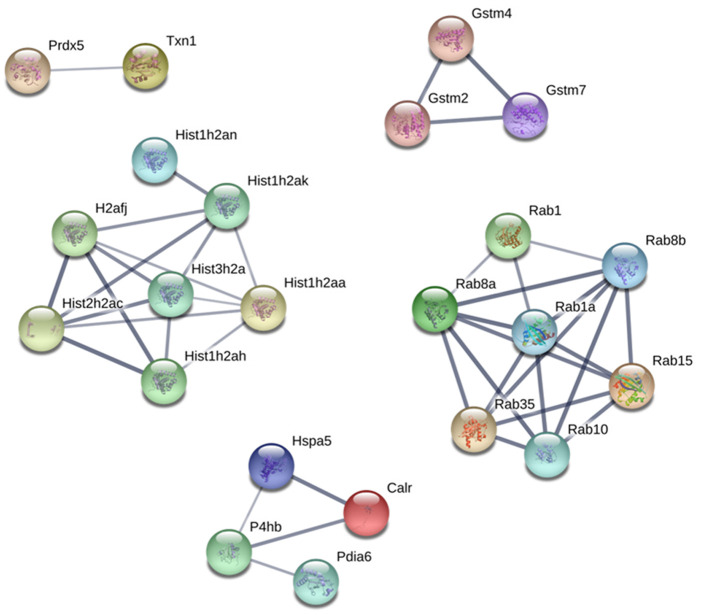
**Protein-protein interaction network between commonly upregulated proteins on both PND 21 and 540.** Interactions of the identified proteins were mapped by searching the STRING database version 9.0 with a confidence cut-off of 0.7. In the resulting protein association network, proteins are presented as nodes that are connected by lines whose thickness represents the confidence level (0.7-0.9).

Protein-Protein interaction (PPI) network analysis demonstrated several clusters for up and downregulated proteins on PND 21 and 540 (Supplementary Figures 1–4). Based on these results, we identified five principal clusters commonly deregulated in both ages: (1) RAB (Ras-related protein) 1, RAB10, RAB15, RAB1A, RAB35, RAB8A, AND RAB8B; (2) H2AFJ (Histone H2A.J), HIST1H2AA, HIST1H2AH, HIST1H2AK, HIST1H2AN, HIST2H2AC, and HIST3H2A; (3) GSTM2 (glutathione S-transferase Mu 2), GSTM4 and GSTM7; (4) CALR (calreticulin), HSPA5 (heat shock protein family A member 5), P4HB (protein disulfide isomerase-4), PDIA6 (Protein Disulfide Isomerase Family A Member 6); (5) PRDX5 (peroxiredoxin-5) and TXN1 (thioredoxin 1). The cluster identified in commonly downregulated proteins on PND 21 and 540 was composed of HBA1 (hemoglobin Subunit Alpha 1), HBA-A2, HBB, and HBE1 (Figure 8).

### *In silico* analysis confirmed the relationship between differentially expressed proteins (DEP) and PCa in both rodent model and human samples

**Figure 9:**
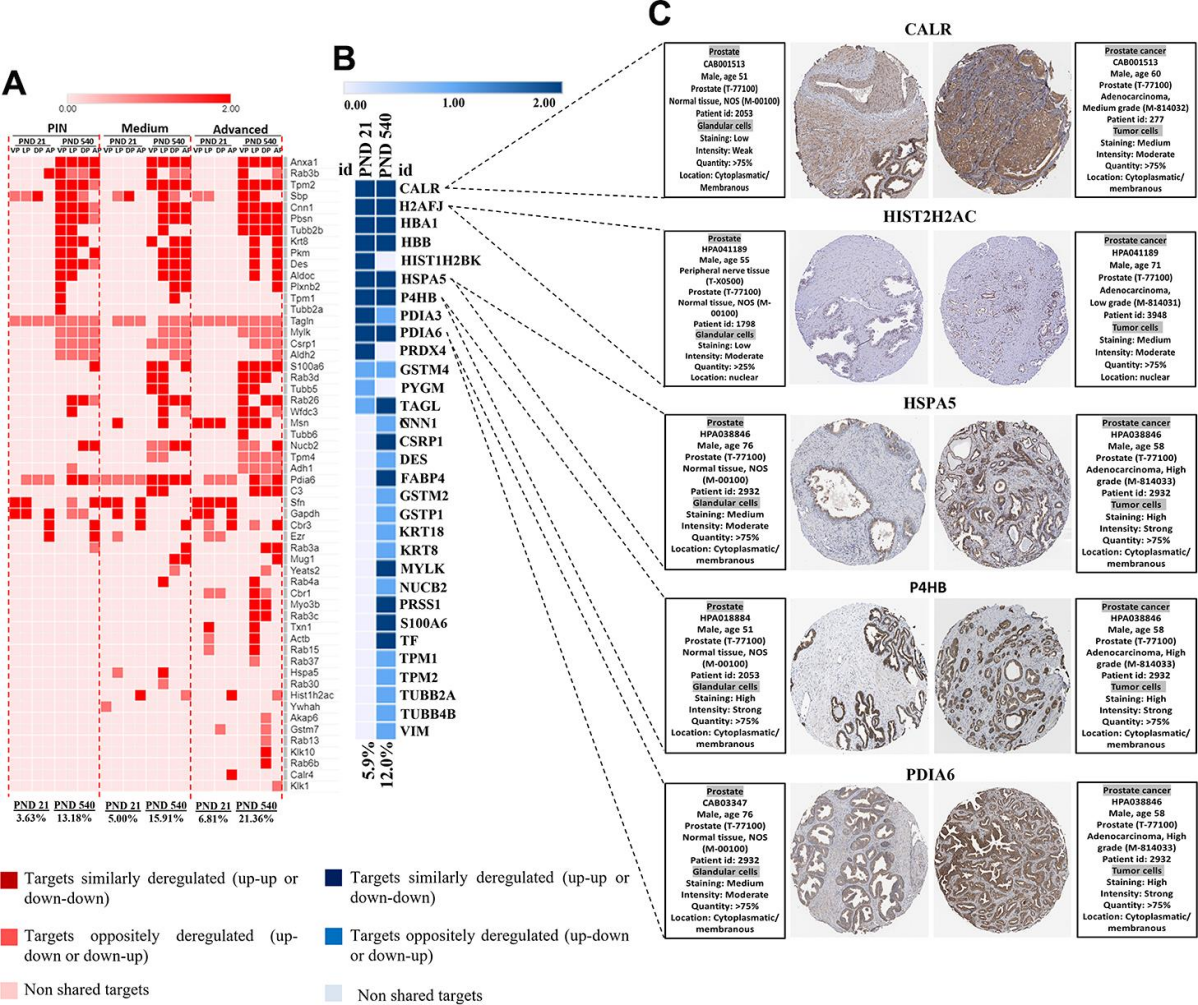
(**A**) Heatmap showing the commonly differentially targets from our set of DEP and RNA-seq data from ventral, dorsal, lateral, and anterior prostate lobes in the mice model of PCa (PB-Cre/PtenloxP/loxP). The percentage of commonly deregulated targets increases as the prostatic disorders worsen (PIN to Medium to Advanced PCa). (**B**) The commonly deregulated targets between our DEP and those extracted from RNA-seq data by GEPIA. The percentage of commonly deregulated targets increases in the prostate of older offspring. (**C**) Immunostaining of normal and prostate tumor samples for five commonly upregulated targets in our samples and GEPIA database (http://gepia.cancerpku.cn/) using immunohistochemical data available at the Human Protein Atlas database (https://proteinatlas.org/). PND: Postnatal day; VP: Ventral prostate; LP: Lateral prostate; DP: Dorsal prostate; AP: Anterior prostate PIN: Prostate intraepithelial neoplasia; PCa: Prostate cancer.

To give further insights into the role of maternal malnutrition on prostate carcinogenesis, we compared our set of DEP with data from a transgenic PCa mouse model and data from The Cancer Genomic Atlas (TCGA) patients with PCa taken from Gene Expression Profiling Interactive Analysis (GEPIA). In the PB-Cre/Pten^loxP/loxP^, we identified a set of DEP commonly expressed in our samples, mainly on PND 540 and in the prostatic tumors in all prostatic lobes. Interestingly, the percentage of commonly deregulated targets between our samples and those from the PB-Cre/Pten^l oxP/loxP^ model increased with aging (from PND 21 to 540) and with the aggressiveness of prostatic lesions (PND 21: PIN 3.6%; medium 5.0%; advanced 6.8% and PND 540: PIN 13.1%; medium 15.9%; advanced 21.3% (Figure 9A). Similar results were obtained when our set of DEP was compared with data from patients with PCa (from 5.9% on PND 21 to 12.0% on PND 540) (Figure 9B). We also identified six proteins (CALR, HIST2H2AC, HSPA5, P4HB, and PDIA6) in the HPA database that showed increased immunostaining in PCa tumor tissue, while low or not detected in normal prostate tissue (Figure 9C). These results highlight the involvement of maternal malnutrition in the deregulation of proteins involved in prostate tumors.

### Experimental validation of CALR as upregulated protein in offspring exposed to maternal malnutrition

**Figure 10:**
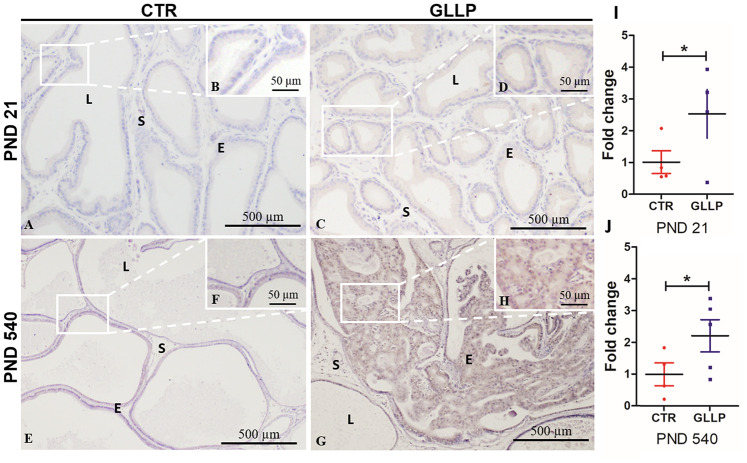
Representative immunohistochemistry reaction for Calreticulin (CALR) in the VP lobes from the CTR and GLLP groups on PND 21 (**A**–**D**) and 540 (**E**–**H**). RT-qPCR reaction for CALR in the VP lobes from CTR and GLLP groups on PND 21 (**I**) and 540 (**J**). CTR = control; GLLP = gestational and lactational low protein; PND = postnatal day. Data are expressed as fold change {plus minus} SD. Asterisks (*) means the statistical difference between experimental groups with p < .05. Scale bar: 500 μm, and detail 50 μm.

Based on the proteomic data (Supplementary File 1) and *in silico* analysis, we employed immunohistochemical and RT-qPCR analyses to validate the CALR as an upregulated target in the offspring VP born to dams fed with LPD in both PND 21 and 540. Immunostaining for CALR was more evident in the GLLP group on both PND 21 (Figure 10C and 10D) and PND 540 (Figure 10G, 10H) compared to the CTR group (Figure 10A, 10B, 10E, 10F) mainly in areas of carcinoma in situ. RT-qPCR confirmed the upregulation of CALR gene expression in the GLLP group at both ages (Figure 10I, 10J).

## DISCUSSION

Although maternal exposure to adverse conditions has been identified as an essential window for the development of non-communicable diseases in the progeny, there is a growing body of evidence highlighting malnutrition during early life as a key environmental risk factor for the developmental origin of some types of diseases such as some types of cancer, including breast and PCa in offspring [12–16, 18, 28–30]. However, little data is supporting the molecular pathways associated with early life carcinogenesis and understanding this aspect may be crucial to identifying and perhaps modulating molecular pathways involved in the developmental origin of diseases, especially in those more vulnerable populations, who have limited access to more expensive food components, such as proteins [31].

Consistent with our previous results [16, 32], the set of deregulated proteins identified in a mass spectrometry analysis was associated with the molecular mechanism classically recognized as a potent regulator of development, maintenance of tissue homeostasis, and disease. FGFs and EGFs act to regulate glandular morphogenesis, cell proliferation, and differentiation and have secretory functions not only during development but also during neoplastic transformation and tumor progression [33–38]. The Hippo signaling pathway also plays a crucial role in the control of organ size, branching morphogenesis, and tissue homeostasis by regulating cellular mechanisms such as cellular polarity, cell-cell contact, and cytoskeleton organization [39, 40]. The enrichment of molecular pathways related to the endoplasmic reticulum function, such as the metabolism of xenobiotic, chemical carcinogenesis, endocytosis, in addition to cell adhesion, longevity, and apoptosis also highlights the involvement of these cellular and molecular mechanisms in prostatic carcinogenesis [41–45]. The maintenance of these processes is crucial for glandular homeostasis. As a consequence, the breakdown of these molecular mechanisms may also interfere with cell-cell adhesion and metabolism, the maintenance of barriers between the blood and the epithelial and stromal compartments, in addition to interfering within the dynamics of cellular differentiation, proliferation, and migration, all mechanisms involved in the initial stages of carcinogenesis [46–49]. Our data also confirmed the involvement of the estrogen signaling pathway in maternal malnutrition inducing early life prostate carcinogenesis in rat offspring. Previous experimental evidence has demonstrated that early life exposure to exogenous estrogenic compounds, such as BPA and Phthalates, may epigenetically reprogram prostate developmental biology, lead to prostate carcinogenesis with aging [16, 50].

Protein-protein interactions play a crucial role in the control of cellular functions, signal transduction, and metabolism; as such, understanding these interactions may help us to identify molecular mechanisms involved in maternal malnutrition-induced prostatic disorders. The RAB family of proteins, identified by a PPI network analysis, belongs to the RAS (rat sarcoma) superfamily of small GTPase. RAB comprises a family of 66 members (the number of RAB-GTPases is conserved from yeast to humans) [51, 52], which function as molecular regulators essential for the localization and function of the membrane and secretory proteins such as hormones, growth factors, and their membrane receptors. As such, RAB activates several signaling pathways, including the MAPK pathway involved in cell growth and proliferation [53] and the PI3K/AKT/mTOR pathway that stimulates protein synthesis, cell growth, and inhibits apoptosis [54]. Altered expression and the activity of RAB members have been implicated in the development of several disorders, ranging from neurological disorders to diabetes [55]. Aberrant expression of RAB proteins has also been described in multiple cancers, such as lung, brain, and breast. RAB 35, which appeared to be deregulated in our study, can act as an oncogene [56]. The GTPase-deficient RAB35 mutant (RAB35Q67L) activates the PI3K signaling pathway independently of growth factor stimulation and suppresses apoptosis in human embryonic kidney HEK293E cells [57]. The cluster formed CALR, HSPA5, P4HB and, PDIA6 is a potential indicator of maternal malnutrition on the endoplasmic reticulum dysfunction in the prostate of offspring since they act as the fundamental molecular machinery for correct protein folding and Ca^2+^ homeostasis. In a mouse model of caloric restriction (CR), Schafer et al. [58] compared the influence of CR on the hippocampus at younger-adult and older-adult time points and identified the upregulation of HSPA1B, HSPA5, PDIA4, PDIA6, and CALR. Other authors have also associated the deregulation of these proteins in several types of cancer, including breast carcinoma, hepatoma cells, non-small cell lung cancer, and glioma [59–63]. Although epigenetic modifications of histones, such as histone lysine methylation and demethylation, histone lysine acetylation and deacetylation have been implicated in the modulation of gene expression in the physiological and pathological conditions [64, 65], deregulation of histone expression itself has also been described in several types of malignancies. Xie et al. [66] demonstrated upregulation of hub genes formed by HIST1H1B, HIST1H2AJ, HIST1H2AM, HIST1H2BI, HIST1H2BO, HIST1H3B, HIST1H3F, HIST1H3H, HIST1H4C, and HIST1H4D in breast cancer, indicating that higher expression of these histones was associated with poor overall survival, relapse-free survival, and distant metastasis-free survival. Interestingly, we also observed an increased expression of HIST1 gene members (HIST1H2AA, HIST1H2AH, HIST1H2AK, HIST1H2AN) in the VP of both young and older rats exposed to maternal LPD. This result highlights the potential involvement of the upregulation of histone proteins on prostatic disorders.

Another cluster is formed by enzymes acting in response to oxidative stress as GST, PRDX, and TXN1. The superfamily of GSTs acts in several mechanisms of cellular detoxification, resistance to anticancer drugs, pollutants, and chemicals [67], and the overexpression of GSTs is also associated with the presence of an inflammatory process [68]. It has been demonstrated that low expression of GSTs increases reactive oxygen species in spermatozoids, leading to a degradation of the plasma membrane and a loss of sperm viability [69–71]. In the prostate gland, GSTs are mainly expressed in the basal cells [72], and their overexpression is associated with epithelial disorders [73], DNA oxidation, and methylation [74, 75]. PRDX-5 is known to act as a redox sensor in the cytosol and several cellular compartments, and silencing it makes the cell more susceptible to DNA damage and apoptosis [76]. In gastric cancer, overexpression of PRDX5 alters the epithelial to mesenchymal transition (EMT) mechanism, with a poor prognosis for patients [77] being correlated. TXN recycles oxidized PRDXs, and this function is essential to balance intracellular oxidative stress [78, 79]. The increased expression of TXN in prostate tissue has been positively correlated with the progression of Gleason score in patients with PCa [80], indicating that transformed cells express higher levels of Trx 1 compared with normal cells. On the other hand, the treatment of prostate cancer cells with natural bioactive compounds reduces TXN expression, collaborating with the apoptosis of these cells [81]. Thus, the high expression of TXN in the prostate of maternal LPD offspring could be related to the development of carcinoma in situ, as observed in older rats.

Considering that groups of interacting proteins are deregulated in both younger and older undernourished offspring, it is possible that histones and RAS-GTPase families and proteins related to oxidative stress, besides those involved with endoplasmic reticulum metabolism and function, may participate in the long-term effect of maternal malnutrition on the prostate of offspring. These results become more relevant with the identification of several of these proteins in patients and mice model of PCa by *in silico* analysis.

## CONCLUSIONS

In the present study, we show that maternal exposure to low protein diet deregulated molecular pathways involved in prostate development early in life, which may act as risk factors for prostate carcinogenesis with aging. Estrogenic signaling pathways, endoplasmic reticulum functions related to detoxification, energy metabolism, and molecular sensors of protein folding and Ca^2+^ homeostasis, besides histone, and RAS-GTPase family of proteins appear to be involved in this process. Knowledge of these factors may raise discussions regarding the role of maternal dietary intervention as a favorable public policy for the lifelong prevention of chronic diseases.

## MATERIALS AND METHODS

### Animals and experimental design

The detailed experimental design is described by Santos et al. [16]. Briefly, after the determination of pregnancy on gestational day 1 (GD1), pregnant rats were distributed into two experimental groups (n=6/group): Control (CTR): dams fed a normal protein diet (17% protein) and gestational and lactational low protein (GLLP): dams fed a low protein diet (LPD) during gestational and lactational periods. Normal and LPD diets were provided by PragSoluções (PragSoluções, SP, Brazil). All diets were isocaloric and normosodic (Supplementary Table 1). The male offspring were euthanized on a postnatal day (PND) 21 (weaning) (n=12/group) and PND 540 (n=12/group). The offspring, which were euthanized on PND 540, had free access to a normal protein diet after weaning until the end of the experiment. The animals were euthanized by an overdose of anesthesia (ketamine/xylazine) followed by decapitation, weighing, and the blood and ventral prostate (VP) were collected and processed by a different analysis as described below. The body weight, and VP weight, and hormonal levels were analyzed using a Student t-test, and statistical differences were considered when p < 0.05. The animal procedures were approved by the Biosciences Institute/UNESP Ethics Committee for Animal Experimentation (Protocol #573) following the ethical animal research principles and the Brazilian legislation established by the Brazilian Council of Control in Animal Experimentation.

### Hormone analysis

Blood samples from offspring (n=12/group) were centrifuged (2400 g for 20 minutes), and sera were used to determine the concentrations of estrogen (17β-estradiol, Monobind®, 4925-300 CA, USA sensitivity: 6.5 pg/mL) and testosterone (17β-hydroxy-4-androstene-3-one, Monobind®, 3725-300A, CA. sensitivity: 0.038 ng/mL). The hormonal qualifications were determined in 96-well plates using the ELISA plate reader (Epoch™, Biotek Instruments, VT, USA) following the manufacturers' protocol.

### Selection of prostate samples for mass spectrometry analysis

In a previous study, Santos et al. [16] demonstrated that maternal exposure to LPD induced a delay in prostatic growth on PND 21, which was associated with prostate carcinogenesis in older rats on PND 540. Slides of the left VP lobes (n=3/group) were stained with hematoxylin-eosin (HE) and analyzed using a Leica DMLB 80 microscope To exemplify the histological characteristics of the VP lobes from the CTR and GLLP groups on PND 21 and 540. Based on these results, the contralateral right VP lobes (n=3/group) from each group were submitted to mass spectrometry.

### Immunohistochemistry

Histological sections of 5 μm (n = 6 per group) were processed as described by Santos et al. [16]. After the initial steps, the slides were boiled for 30 min in 10 mM sodium citrate solution (pH 6.0) for antigen retrieval. Prostatic sections were blocked in 5% nonfat milk diluted in phosphate-buffered saline (PBS) and incubated with anti-Calreticulin antibody (ab2908) specific primary antibody overnight at 4°C. Slides were washed in PBS and incubated for one hour at room temperature in horseradish peroxidase (HRP)-conjugated secondary antibody. The slides were washed, and the reaction was developed using 3,3′-Diaminobenzidine (DAB, Sigma) and counterstained with hematoxylin for 30 seconds. The reactions were analyzed using a Leica DMLB 80 microscope.

### RT-qPCR

Prostate samples (n= 6 per group) from all experimental groups on PND 21 and PND 540 were used to total RNA extraction using TRIzol® Reagent (ThermoFisher aScientific) according to the manufacturer's recommendations. RNA integrity was evaluated by capillary electrophoresis using a 2100 Bioanalyzer (Agilent). Only samples with an RNA integrity number (RIN) ≥ 7.0 were used. The extracted RNA was treated with DNase I (Amplification Grade; ThermoFisher Scientific). The synthesis of cDNA was performed using a High Capacity cDNA Archive Kit (ThermoFisher Scientific) according to the manufacturer's guidelines. Expression levels of CALR mRNA were measured by RT-qPCR using the QuantStudio™ 12K Flex Real-Time PCR System (Thermo Fisher Scientific). All qPCRs performed were compliant with the Minimum Information for Publication of Quantitative Real-Time PCR experiments (MIQE) guidelines [82]. The cDNA samples were amplified using SYBR® Green Master Mix (ThermoFisher Scientific), and specific primers were synthesized by Invitrogen to the CALR gene, forward: GCCAGACACTGGTGGTACAGTTC reverse: CGCCCCCACAGTCGATATT. Relative quantification of expression was performed by the 2^−ΔΔCt^ method [83] using DataAssistTM v3.01 software (Thermo Fisher Scientific). According to the expression stability among all samples, the reference gene GUSB (β-glucuronidase) and GAPDH (glyceraldehyde 3-phosphate dehydrogenase) were used to normalize mRNA expression.

### Mass spectrometry

The mass spectrometry protocol was based on a previous study by Gabriel Kuniyoshi et al. [84], Dionizio et al. [85], and Da Silva-Gomes et al. [86], with modifications. Briefly, protein extraction was carried out by homogenizing three VPs lobes from each experimental group on PND 21 and 540 in extraction buffer containing 0.01 M Tris-HCl, 0.005 M phenylmethylsulfonyl fluoride, 1% protease inhibitor, 0.065 M dithiothreitol, 8 M urea (in a proportion of 30mg tissue/100 μg buffer). The homogenate was vortexed for 2-3 min and centrifuged for 15 min at 9,690 g and 4°C. The supernatant was recovered, and the total protein was quantified by Bradford assay using the BSA standard [87]. Samples were grouped to constitute three pools of 50 μg proteins each in a total of 50 μL (1 μg/μL). Next, the samples were incubated for 60 min at 37°C with 10 μL of 50 mM ammonium bicarbonate and 25 μl of 0.2% surfactant solution, followed by incubation with 2.5 μL of 0.1 M dithiothreitol for 40 min at 37°C. Carbamidomethylation was performed with 2.5 μL of 0.3 M iodoacetamide, incubated for 30 min at room temperature, and protected from light. Then, samples were subjected to proteolytic digestion overnight at 37°C using 0.05 μg/μL trypsin diluted in 0.05 M Ammonium bicarbonate, followed by incubation with 10 μL trifluoroacetic acid 5% for 90 min at 37°C. The samples were centrifuged at 14,000 RPM 4ºC for 30 minutes. After this step, the samples were desalted using Sep-Pak Vac C18 (Waters Manchester, UK) columns, reduced in a concentrator, and maintained at -20°C until the time of analysis by mass spectrometry.

The analysis of the tryptic peptide was performed using the nanoACQUITY UPLC system (Waters, Manchester, UK) coupled to a Xevo Q-TOF G2 mass spectrometer (Waters, Manchester, UK) equipped with nanoACQUITY HSS T3, analytical reverse-phase column (75μmX150 mm, 1.8μm particle size, Waters) previously equilibrated with 7% of mobile phase B (100% ACN + 0.1% formic acid). The peptides were separated by a linear gradient of 7-85% mobile phase B for 70 min at a flow rate of 0,35 μL/min, and the column temperature was maintained at 45°C. The MS was operated in positive ion mode, with a data acquisition time of 75 min. The data obtained were processed using the software Protein Lynx Global Server (PLGS) version 3.03 (Waters Co., Manchester, UK). Protein identification was obtained using an ion count algorithm incorporated into the software. The data obtained were searched in the database of the species *Rattus norvegicus* downloaded from the UniProt catalog (Universal Protein Resource) in December 2017 (https://www.uniprot.org/). Differentially expressed proteins (DEP) between experimental groups were obtained using PLGS software, considering p < 0.05 for downregulated proteins and p > 0.95 for upregulated proteins.

### Functional annotation analysis

KOBAS 3.0 (http://kobas.cbi.pku.edu.cn/) was used to determine the enrichment pathways related to our DEP in the KEGG (https://www.genome.jp/kegg/) and PANTHER (http://pantherdb.org) databases. The cut-off criterion used was an adjusted p-value < 0.05. Also, we used the Ligand Perturbation database from the Enrichr tool (http://amp.pharm.mssm.edu/Enrichr) to compare the set of DEP from those extracted from GEO comparing human or mouse cells before and after treatment with endogenous ligands. We used the top 10 most enriched terms with a p-value < 0.05 [88]. The STRING tool (http://string-db.org/) was used to construct the protein-protein interaction (PPI) network associated with our DEP by searching neighbor interactors with our imputed proteins. To avoid false positive interactions, we selected a high confidence score (0.7), associated with experiments and a database as two stringent evidence channels [89].

### Relevance of deregulated proteins in human and mouse model of PCa: *in silico* validation

To give further insights into the relationship between maternal malnutrition and PCa, we compared our set of DEP with RNA-seq data from a transgenic mice model for PCa: PB-Cre/Pten^loxP/loxP^. In this study, Jurmeister et al. [90] described data from the RNA-seq of four prostate lobes (ventral, anterior, dorsal, and lateral) at different stages of tumorigenesis: low-grade prostate intraepithelial neoplasia (PIN), medium-stage tumors (Medium) and advanced-stage tumors (Advanced). The dataset was downloaded from the NCBI Gene Expression Omnibus (https://www.ncbi.nlm.nih.gov/geo/), accession number GSE94574. We considered differentially expressed genes: < -1.3 Log2FC > 1.3, adjusted p-value < 0.05. We also identified differentially expressed genes between normal from Genotype-Tissue Expression (GTEx) with 221 patients/samples and PCa human samples extracted from RNA-seq data using Prostate Adenocarcinoma (TCGA, PanCancer Atlas) with 488 patients/samples data analyzed using the GEPIA database (Gene Expression Profiling Interactive Analysis) (http://gepia.cancer-pku.cn/) [91]. We considered differently expressed genes with < -1 Log2FC > 1 and q-value < 0.05. This set of genes was compared with our DEP to identify possible molecular mechanisms shared by our samples and those from human PCa. Additionally, the commonly upregulated proteins identified in our sample and RNA-seq of prostatic tumor samples identified by GEPIA were submitted to The Human Protein Atlas (HPA) (https://www.proteinatlas.org/) database to demonstrate the distribution and localization of these proteins in normal and tumor samples by immunohistochemistry.

### Data representation and analyses

Bar plots were constructed using GraphPad Prism (GraphPad Software). We used the webserver http://bioinformatics.psb.ugent.be/webtools/Venn/ to plot the Venn diagrams. Heat maps were created using the web tool Morpheus [92] (https://software.broadinstitute.org/morpheus), and circus plots were generated in environment R with package 'circlize' [93].

## Supplementary Material

Supplementary Figures

Supplementary Tables

Supplementary File 1

Supplementary File 2
