# Supplementary material for: Identification of potential molecular pathways involved in prostate carcinogenesis in offspring exposed to maternal malnutrition
Source: Aging (Albany NY). 2020 Oct 13;12(20):19954–78. doi: 10.18632/aging.104093 (PMC7655221; doi:10.18632/aging.104093)
Supplement: Supplementary Figures [file aging-12-104093-s001..pdf]

SUPPLEMENTARY FIGURES

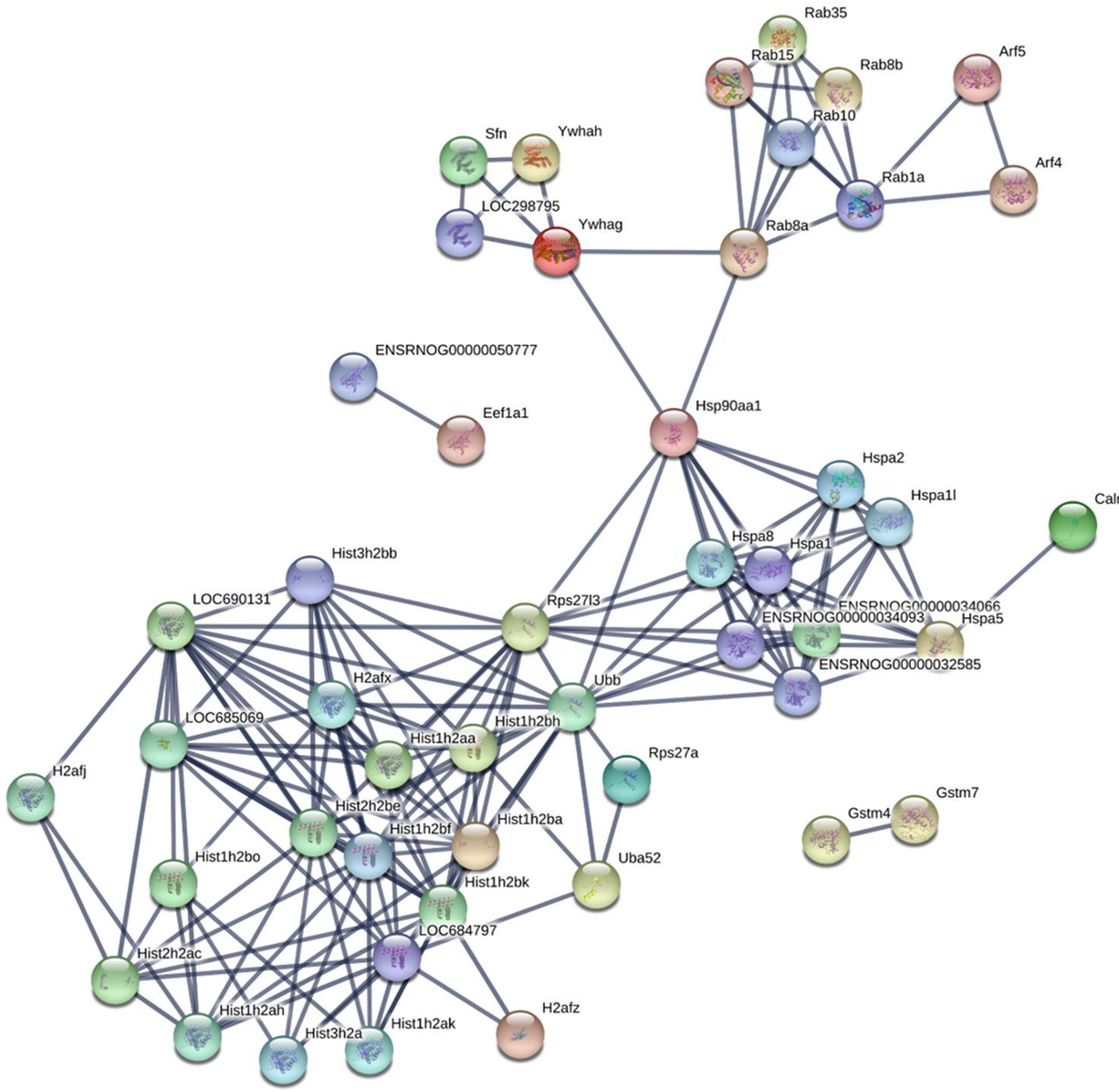

Supplementary Figure 1. Protein-protein interaction network between upregulated proteins on PND 21. Interactions of the identified proteins were mapped by searching the STRING database version 9.0 with a confidence cut-off of 0.7. In the resulting protein association network, proteins are presented as nodes that are connected by lines, whose thickness represents the confidence level (0.7-0.9).

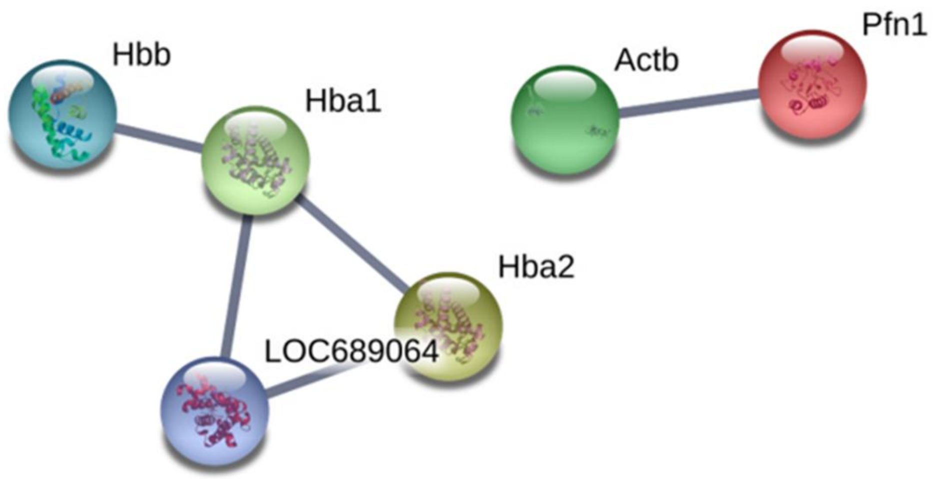

Supplementary Figure 2. Protein-protein interaction network between downregulated proteins on PND 21. Interactions of the identified proteins were mapped by searching the STRING database version 9.0 with a confidence cut-off of 0.7. In the resulting protein association network, proteins are presented as nodes that are connected by lines whose thickness represents the confidence level (0.7-0.9).

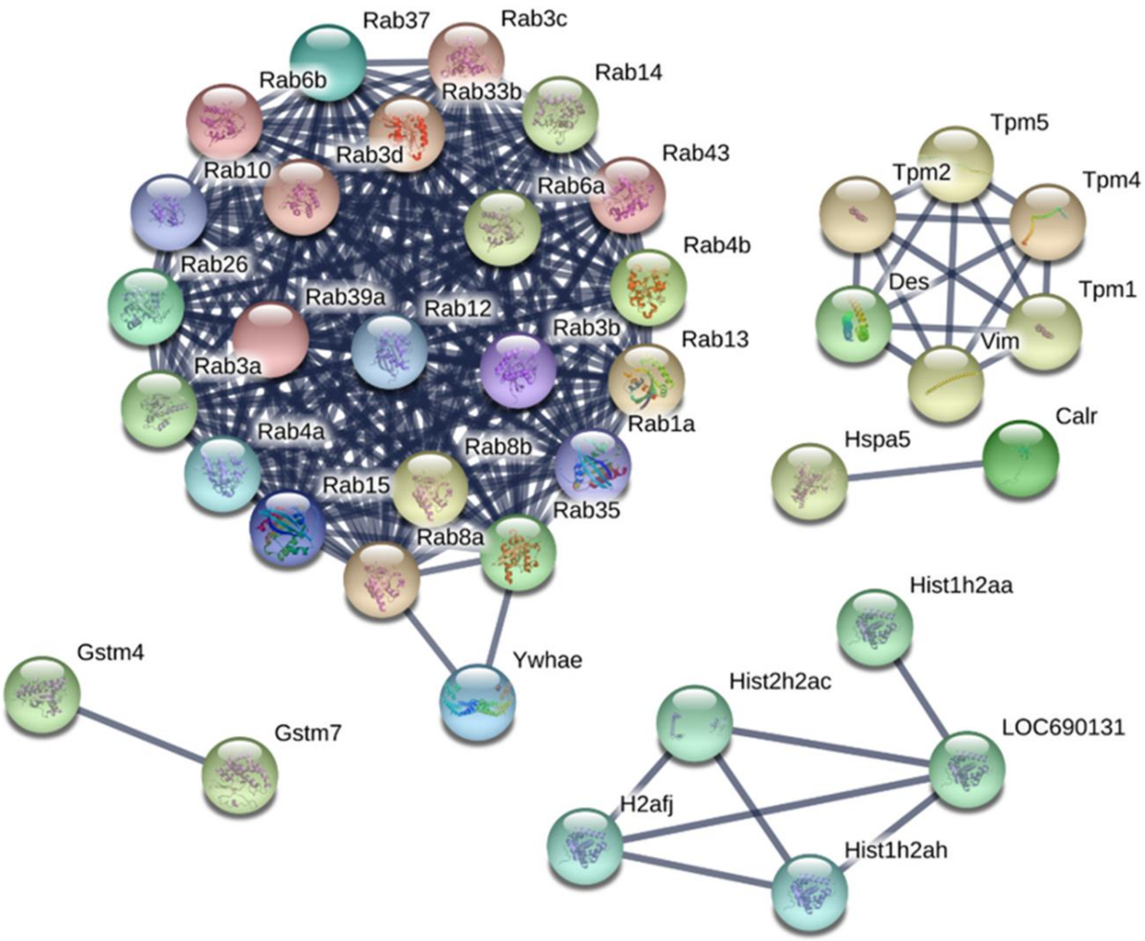

Supplementary Figure 3. Protein-protein interaction network between upregulated proteins on PND 540. Interactions of the identified proteins were mapped by searching the STRING database version 9.0 with a confidence cut-off of 0.7. In the resulting protein association network, proteins are presented as nodes that are connected by lines, whose thickness represents the confidence level (0.7-0.9).

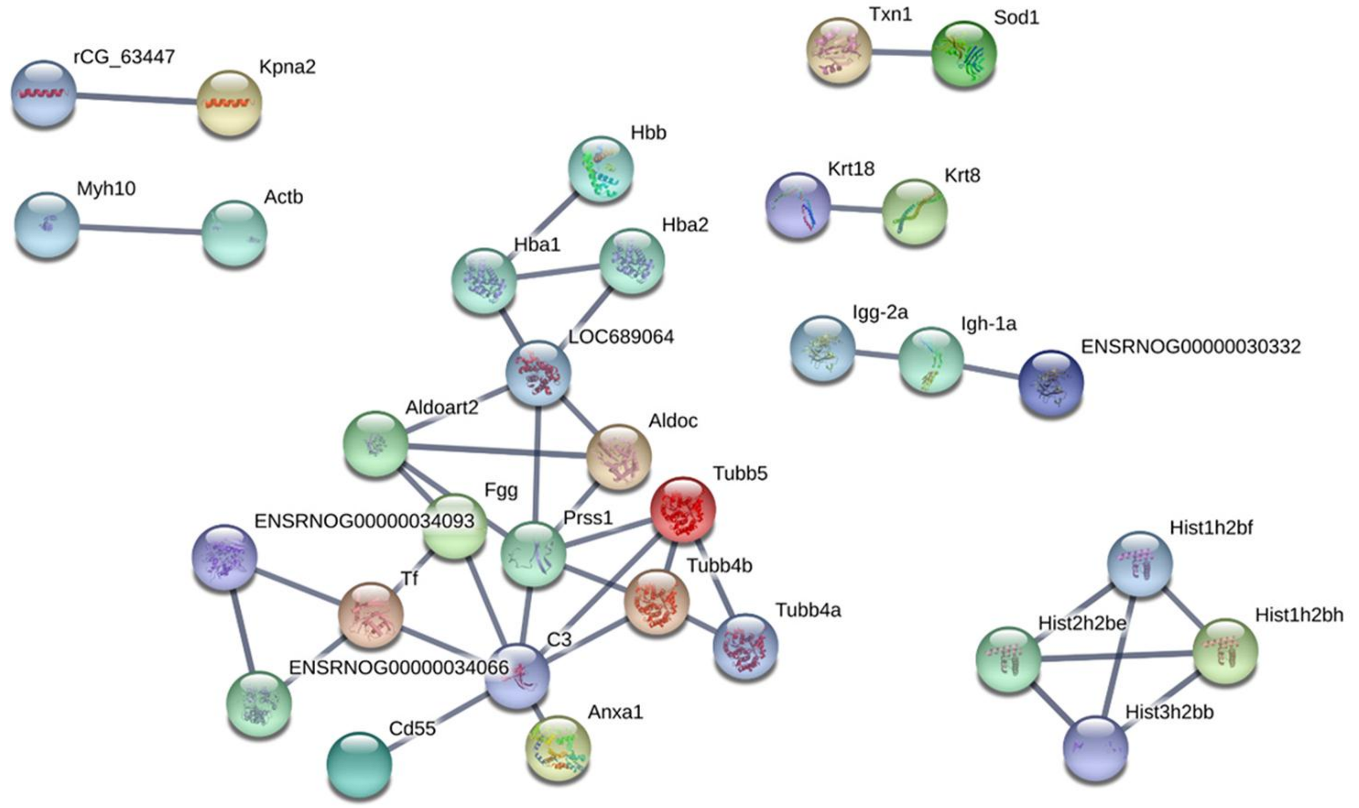

Supplementary Figure 4. Protein-protein interaction network between downregulated proteins on PND 540. Interactions of the identified proteins were mapped by searching the STRING database version 9.0 with a confidence cut-off of 0.7. In the resulting protein association network, proteins are presented as nodes that are connected by lines, whose thickness represents the confidence level (0.7-0.9).
