# Supplementary material for: Identification of potential molecular pathways involved in prostate carcinogenesis in offspring exposed to maternal malnutrition
Source: Aging (Albany NY). 2020 Oct 13;12(20):19954–78. doi: 10.18632/aging.104093 (PMC7655221; doi:10.18632/aging.104093)
Supplement: Supplementary Tables [file aging-12-104093-s002..pdf]

SUPPLEMENTARY TABLE

Supplementary Table 1. Composition of the control (CTR) (AIN-76A) and low protein diet (LPD) (AIN-93).

Ingredients	Normal (CTR) diet (17% of protein) g/Kg	Low protein diet (6% of protein) g/Kg
Casein (84% protein)	202	71.5
Starch	397	480
Dextrin	130.5	159
Sucrose	100	121
L-cystine	3	1
Fiber of pH 101 or pH 102 (microcellulose)	50	50
Soyoil	70	70
Mixtureofvitamins AIN93G*	10	10**
Mixtureofsalts AIN93G*	35	35***
Choline hydrochloride or Choline bitartrate	2.5	2.5

* To know the detailed composition of the salt and vitamin mix, see REEVES et al., 1993. The diet is elaborated by the company PragSoluções (PragSoluções, Jaú, SP, Brazil).
